# Development of non-communicable disease risk factors in Finland: projections up to 2040

**DOI:** 10.1177/14034948221110025

**Published:** 2022-07-26

**Authors:** Hanna Tolonen, Jaakko Reinikainen, Zhi Zhou, Tommi Härkänen, Satu Männistö, Pekka Jousilahti, Laura Paalanen, Annamari Lundqvist, Tiina Laatikainen

**Affiliations:** Department of Public Health and Welfare, Finnish Institute for Health and Welfare, Finland

**Keywords:** Smoking, sedentary lifestyle, hypertension, cholesterol, obesity, diabetes, projection

## Abstract

**Background:**

Non-communicable diseases are a major cause of mortality and morbidity worldwide. They share the same behavioural risk factors (smoking, sedentary behaviour, alcohol consumption and an unhealthy diet), all of which are modifiable risk factors, and biological consequences (hypertension, elevated total cholesterol, obesity and diabetes).

**Methods:**

Using data from a series of cross-sectional health examination surveys conducted among the adult population in Finland from 1997 to 2017, a projection of risk factor development (smoking, leisure time sedentary behaviour, hypertension, elevated total cholesterol, overweight and obesity, and diabetes) up to the year 2040 was made. The projections were estimated using a multiple imputation method.

**Results:**

Smoking prevalence is estimated to continue to decline up to 2040, similar to hypertension and elevated total cholesterol. By contrast, obesity and diabetes will develop unfavourably, with an increase in prevalence. The increase in obesity is mainly due to polarisation – that is, normal-weight people remain of a normal weight, but overweight people tend to gain more weight and become obese. The observed and estimated changes for leisure time sedentary lifestyle were not statistically significant.

**Conclusions:**

**Projections of risk factors for non-communicable diseases are needed to guide public health policies and programmes, decision-making and the allocation of health care resources for prevention and care. In Finland, favourable developments have been seen in many of the risk factors, but obesity and diabetes show unfavourable development. There is a need to continue regular, systematic monitoring of the development of risk factors through health examination surveys and to set national goals and programmes to tackle the existing problems.**

## Introduction

Non-communicable diseases (NCDs) are a major cause of mortality and morbidity worldwide. The situation in Finland is similar to other high- and middle-income countries: in 2020, the leading causes of death were diseases of the circulatory system, followed by neoplasms and memory disorders [1]. For morbidity, most of the primary health care visits, based on ICD-10 codes, were related to hypertension, back pain and upper respiratory infections, followed by other soft tissue disorders and type 2 diabetes [2].

NCDs – for example, cardiovascular disease (CVD), cancer and chronic respiratory disease – share the same behavioural risk factors (smoking, sedentary behaviour, harmful use of alcohol and an unhealthy diet) and biological consequences (hypertension, elevated total cholesterol, obesity and diabetes, which is also an NCD). These are modifiable risk factors, meaning that disease risk can be reduced with a healthy lifestyle. By contrast, we cannot change our sex, age, ethnicity nor family history of disease (genetic background), which are also known risk factors for many NCDs. The disease burden or distribution of risk factors are not equally distributed between different population sub-groups – for example, differences in socioeconomic status often lead to health inequalities within a population.

Smoking is the leading cause of chronic obstructive pulmonary disease (COPD), lung cancer and an important risk factor for CVD. Lifelong smokers have a 50% probability of developing COPD during their lifetime and 80% of lung cancers could be prevented by eliminating tobacco smoke [3]. A sedentary lifestyle has been associated with a 37% higher risk of CVD mortality compared with a lifestyle including regular physical activity [4].

Hypertension is one of the most common risk factors for CVD. It has been estimated that, worldwide, 54% of cases of stroke and 47% of cases of coronary heart disease could be attributed to high blood pressure. Only about half of this burden is due to clinical hypertension and rest is due to milder levels of high blood pressure [5, 6]. Elevated total cholesterol is known to increase the risk of CVD. The risk ratio for coronary heart disease has been estimated to increase by 20% for each 1 mmol/l increase in total cholesterol [7]. There is much epidemiological evidence that obesity has a causal effect on diabetes, CVD (ischaemic heart disease and ischaemic and haemorrhagic stroke), several cancers and musculoskeletal disorders, such as osteoarthritis and low back pain [8].

The development of NCD risk factors has been followed regularly in Finland since the early 1970s using cross-sectional health examination surveys. The prevalence of current smoking, hypertension and elevated total cholesterol have been decreasing in recent decades, whereas obesity and diabetes have substantially increased. The prevalence of a sedentary lifestyle has remained stable, with a slight increase [9,10]. At the same time, the population in Finland has aged and changes in population structure are expected to continue in the future [11].

Our aim was to prepare projections up to year the 2040 in Finland for six modifiable NCD risk factors (smoking, sedentary lifestyle, hypertension, high total cholesterol, obesity and diabetes).

## Methods

### Data

We used data from five cross-sectional health examination surveys conducted every five years between 1997 and 2017 in Finland: the FINRISK 1997–2012 surveys [12] and the FinHealth 2017 Study [13]. To ensure the comparability of the target populations between the survey years, the available data were restricted to five geographical study areas used in the FINRISK surveys and the common age range of 25–64 years. The six NCD risk factors available in all included surveys were chosen: current smoking, leisure time sedentary lifestyle, hypertension, elevated total cholesterol, obesity and diabetes. The indicators used were defined using data from self-reported questionnaires, objective measurements and the results of laboratory analyses of blood samples.

Smoking status was defined as current smoking from the questions ‘Have you ever smoked regularly’ and ‘When was the last time you smoked’. Leisure time sedentary lifestyle was assessed by the question ‘How much do you exercise and stress yourself physically in your leisure time?’. There were four response options, of which the most inactive one (‘I read, watch TV, and work in the household with tasks which do not make me move much and which do not physically tax me’) was defined as leisure time sedentary lifestyle.

Hypertension was defined as systolic blood pressure ⩾140 mmHg and/or diastolic blood pressure ⩾90 mmHg and/or the reported use of medicine for high blood pressure in the last seven days. Total cholesterol was defined to be elevated if it was ⩾5 mmol/l and/or the person had reported current use of prescription medicine to lower their cholesterol level.

Body mass index (BMI) categories were defined from measured height and weight as normal weight (BMI <25 kg/m^2^), overweight (BMI ⩾25 kg/m^2^ but <30 kg/m^2^) and obese (BMI ⩾30 kg/m^2^). The proportion of underweight was so small (0.8%) that it was combined with the normal-weight category. Diabetes (any type) was defined based on self-reported current use of prescription medicine for diabetes and/or having glycohemoglobin (HbA1c) ⩾48 mmol/mol. HbA1c measurements were not available for the FINRISK 1997 and 2007 surveys.

Other variables used as covariates were sex, age, area and survey year obtained from the sampling frame and self-reported marital status and educational level from the survey questionnaire. Age and survey year were considered as continuous variables, area had five categories, educational level and body weight had three categories and the all variables were binary. Table I presents the sample sizes and response rates for the questionnaire and participation rates for health examination by sex and survey year. In general, people who decided to respond to the survey questionnaire also attended the health examination. More detailed information about item response rates is given in Supplementary Table I, available online.

**Table I.** Sample sizes and response rates for questionnaires and participation rates for health examination from the FINRISK 1997–2012 surveys and the FinHealth 2017 Study for the age group 25–64 years.

		Survey year				
		1997	2002	2007	2012	2017
Original sample size	Men	5000	4999	4000	4000	1635
	Women	5000	5000	4000	4000	1542
Response rate to the questionnaire (%)	Men	67.9	65.0	60.3	56.9	60.6
	Women	72.3	75.3	70.5	66.5	70.0
Participation rate in the health examination (%)	Men	67.9	58.8	56.1	51.5	51.9
	Women	72.3	69.8	65.6	59.7	61.9

This study was performed in line with the principles of the Declaration of Helsinki. All surveys followed the code of ethics in effect at the time of the study. The included surveys had obtained approval from the relevant ethics committees. Written informed consent was obtained from the participants.

### Statistical methods

The population level prevalence of the six NCD risk factors was projected by simulating the individual level values in unobserved samples in the future by multiple imputation – that is, the future health examination surveys were considered as missing data. After the imputation, the predicted risk factor values were pooled to obtain the prevalence. This projection method has been described in more detail elsewhere [14,15]

The unobserved samples of the years from 2020 to 2040 were created to consist of 10,000 participants. The age and sex distributions were matched to the distributions of the national population projections in Finland for the corresponding years [16] to take into account the changing population structure. In addition to these unobserved future samples, missing values (item non-response) in the observed samples were also imput simultaneously. The data frame contained the entire invited samples, so missing values due to both item and unit non-response were imputed.

Multiple imputation was carried out using the mice package [17] in R software. Logistic regression (logreg) and polytomous logistic regression (polyreg) models were used to impute binary and categorical variables, respectively. Age, sex and survey year had no missing values.

Current smoking, leisure time sedentary behaviour, hypertension, elevated total cholesterol, BMI category and diabetes as variables of interest were considered as candidates for predictors in the imputation models. The selection of imputation models for the six risk factors of interest was based on the Bayesian information criterion [18]. The need for non-linear modelling of the survey year was tested using restricted cubic splines as an alternative to linear association [19]. We used Wilson intervals [20] as the prediction intervals of the risk factor prevalence. The number of multiply imputed datasets was 100 and the number of iterations in the chained equations imputation was 10.

## Results

**Figure 1.**
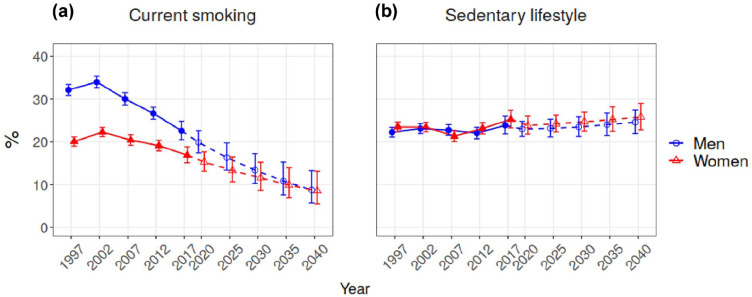
Trends and projections for current smoking and leisure time sedentary behaviour.

**Figure 2.**
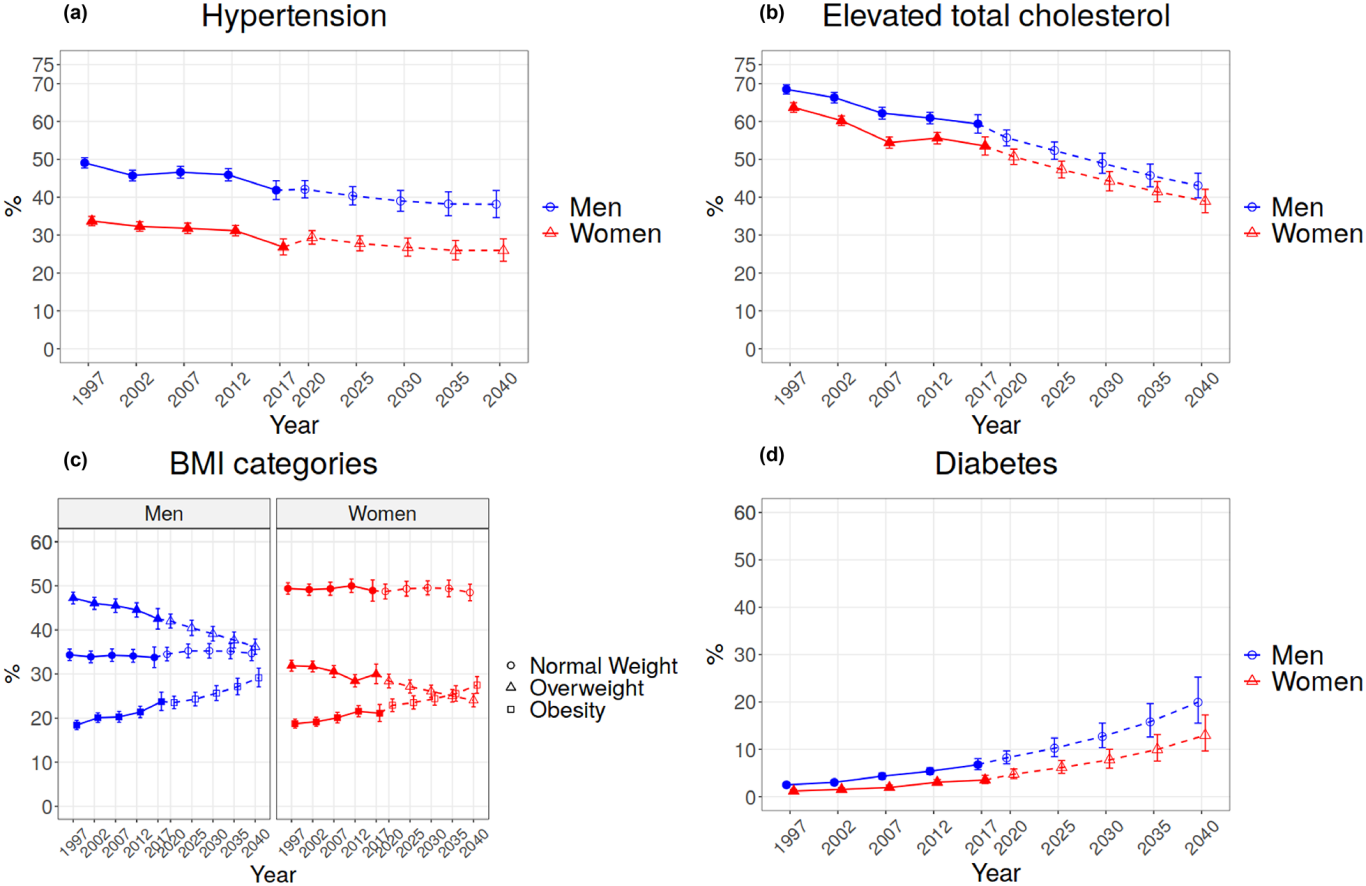
Trends and projections for hypertension, elevated total cholesterol, BMI categories and diabetes.

Figures 1 and 2 show the results for our projections the and absolute and relative changes in relation to the last observed year (2017) are given in Table II.

**Table II.** Absolute (percentage points) and relative (%) changes for 2017.

Risk factor	Sex	Observed change from 1997 to 2017		Observed prevalence in 2017 (%)	Projected change from 2017					
					2025		2030		2040	
		Absolute change (percentage points)	Relative change (%)		Absolute change (percentage points)	Relative change (%)	Absolute change (percentage points)	Relative change (%)	Absolute change (percentage points)	Relative change (%)
Current smoking	Men	–9.6	–29.7	22.6	–6.3	–27.7	–9.2	–40.7	–13.8	–61.2
	Women	–3.2	–15.8	16.9	–3.6	–21.3	–5.3	–31.6	–8.3	–49.3
Leisure time sedentary behaviour	Men	1.6	7.3	23.9	–0.7	–3.0	–0.4	–1.6	0.7	3.0
	Women	1.8	7.6	25.3	–1.0	–4.1	–0.6	–2.4	0.5	2.0
Hypertension	Men	–7.2	–14.7	41.9	–1.5	–3.6	–2.8	–6.8	–3.7	–8.9
	Women	–6.9	–20.4	26.8	0.9	3.5	–0.1	–0.3	–0.9	–3.3
Elevated total cholesterol	Men	–9.1	–13.3	59.4	–7.05	–11.9	–10.40	–17.5	–16.29	–27.4
	Women	–10.2	–16.0	53.4	–6.23	–11.6	–9.30	–17.4	–14.57	–27.2
Obesity	Men	5.3	29.0	23.7	0.5	2.3	1.9	8.0	5.4	22.8
	Women	2.4	12.6	21.1	2.4	11.5	3.3	15.7	6.4	30.3
Overweigh	Men	–4.7	–10.0	42.5	–2.1	–4.8	–3.4	–8.0	–6.3	–14.3
	Women	–1.9	–5.9	30.0	–2.9	–9.5	–3.9	–13.1	–6.0	–19.9
Diabetes	Men	4.3	170.6	6.8	3.5	51.1	5.9	87.6	13.2	194.2
	Women	2.4	196.4	3.6	2.6	73.9	4.2	119.7	9.5	266.6

The prevalence of current smoking has been declining steadily in both men and women since 2002 (Figure 1(a)). In the last 20 years we have seen an almost 30% decrease in current smoking in men and a 16% decrease in women (Table II). This decrease is projected to continue and, by 2040, the prevalence of current smoking for both men and women will be around 9% (Figure 1(a)).

For leisure time sedentary behaviour, stable development was observed until 2012 after which a small increase was seen in 2017 in both men and women (Figure 1(b)). Changes in the last 20 years have been small, with an about 8% increase in both men and women (Table II). The projection for 2040 does not show any significant change in the prevalence of leisure time sedentary behaviour in either men or women, reaching about 25% for both sexes (Figure 1(b)).

A steady decrease was observed for hypertension until 2017 (Figure 2(a)). A notable decrease of 15% for men and 20% women in the last 20 years has been observed (Table II). The projection indicates a modest decrease continuing until 2040, resulting in a prevalence of 38% for men and 26% for women.

The prevalence of elevated total cholesterol has decreased in the last 20 years, by 13% in men and 16% in women (Figure 2(b), Table II). The projections show a continuing decrease for both men and women, resulting in a prevalence of 43% for men and 39% for women by 2040.

For obesity, we can observe an increasing trend in recent decades. The proportion of normal weight remains almost unchanged in both men and women, whereas the prevalence of overweight seems to decrease at the same pace as obesity is increasing (Figure 2(c)). For the BMI categories, observed absolute decrease in the prevalence of overweight (decrease of 5% for men and 2% for women) during the last 20 years is equal to observed increase in the prevalence of obesity (5% for men and 2% for women). In the relative changes, obesity has had steeper increase (29 and 13% for men and women, respectively) than the decrease in overweight (decrease of 10 and 6% for men and women, respectively) (Table II). The projection indicates that obesity will keep increasing and will be 29% for men and 27% for women in 2040 (Figure 2(a)). By 2040, the prevalence of obesity is projected to be almost 23% in men and 30% in women, an increase from 2017 and, at the same time, overweight is projected to have about a 14% decrease in men and a 20% decrease in women (Table II).

For diabetes, a steady increase was observed in the past (Figure 2(d)). The observed absolute changes in diabetes over the last 20 years have been small (4% for men and 2% for women) due to the low prevalence of diabetes in 1997. The relative changes over the same time period have been substantial because the prevalence of diabetes has increased by 170% in men and almost 200% in women (Table II). A substantially steep increase is projected for 2040, resulting in a diabetes prevalence of 20% for men and 13% for women (Figure 2(b)). As a result of the low prevalence in the past, the projected prevalence has a high uncertainty.

## Discussion

Many of the NCDs could be prevented through healthy lifestyles choices by influencing modifiable risk factors (smoking, a sedentary lifestyle and an unhealthy diet) and their biological consequences (hypertension, elevated total cholesterol, obesity and diabetes). Favourable past developments have been observed in Finland for smoking, hypertension and elevated total cholesterol, while substantial increases have been observed in obesity and diabetes. When combining the known history of these risk factors and the projected development of the population structure in Finland, the prevalence of smoking, hypertension and elevated cholesterol will continue to decrease up to 2040. At the same time, obesity and diabetes will continue to increase steeply, whereas leisure time sedentary lifestyle will remain unchanged or increase more slowly.

Previously published projections of risk factor development in Finland have been based only on the observed development of risk factors. These projections were up to 2030 and did not include projected changes in population or missing data within surveys [21–24]. Previous publications have focused on the World Health Organization (WHO) NCD targets and used indicators defined for this purpose [25].

Previous projections for the development of the smoking in Finland [21] were similar, with a continuation of the decreasing pattern among men; our current projection leads to a substantially lower prevalence in both 2030 and 2040. This may be partly due to the different data sources used and the age groups covered in the analysis. A national goal listed in the Finland Tobacco Act 2010 [26] was to reduce the use of tobacco products so that only 5% of the population uses them in 2030. Based on our projections, this target will not be reached by 2030 without marked new measures that to change the direction of future smoking trends. Nevertheless, the projected decrease will contribute to the clear decrease in the incidence of COPD and lung cancer if we assume that 50% of new COPD cases are attributed to smoking and 80% of lung cancer cases could be prevented by not smoking.

For physical activity, previous studies have used sufficient leisure time physical activity (at least three hours per week), which differs from the indicator used here (leisure time sedentary lifestyle) [22]. Based on a previous study, the prevalence of those who do not have a sufficient physical activity level is decreasing, whereas our estimations show that the prevalence of people with leisure time sedentary lifestyle is slightly increasing. Even though a large proportion of people in Finland are physically active, actions are needed to nudge those with a sedentary lifestyle. The direct impact of the projected modest increase in sedentary lifestyle for NCD prevalence would be small, but would contribute to the obesity epidemic and therefore indirectly to the burden of several diseases.

For hypertension, previous projections have shown a similar decreasing pattern to our projections [23]. Previous projections did not include people receiving antihypertensive drugs, which partially explains the higher prevalence in our projections. The proportion of people with hypertension is still projected to be high in 2040 (38% of men and 26% of women). A large proportion of people with hypertension are without treatment or their blood pressure levels are not under control regardless of treatment. Also, too much salt is consumed in the Finnish diet. In 2017, <5% of adults reached the recommended salt intake of ⩽5 g/day [27]. At the same time, the simultaneously increasing prevalence of obesity may partially slow down the projected decrease in hypertension.

There was no previously published projection for elevated total cholesterol in Finland because total cholesterol is not listed as risk factor to be monitored under the WHO NCD targets. Nevertheless, a substantial positive change (i.e. a decrease in total cholesterol levels) has been seen and this decrease is projected to continue into the future. Regardless of this favourable development, the prevalence of elevated total cholesterol is still high and further actions are needed to promote a healthy diet, physical activity and no more than a moderate use of alcohol.

For obesity, previous projections were simple extrapolations based on two time points [24]. Our results showed that the population level distribution of BMI in the last 20 years has not shifted, but has changed its shape. The polarisation of the BMI distribution towards normal weight and obesity can be seen. This might reflect, for example, changes in attitudes on overweight and obesity and body esteem as well as obesogenic environments challenging the deceleration in weight gain. Finland had a national obesity programme in 2012–2018, which aimed to stop the increase in obesity [28]. Based on current developments and projections, further actions are needed to reach the WHO NCD target for obesity [25], which is to stop the increase in obesity. The projected increase in obesity will also affect the projections of diabetes and hypertension. Our model has taken this into account. The promotion of healthy lifestyles with respect to physical activity and a healthy diet are essential.

For diabetes, previous projections were based on the results from two time points [24] and indicated that there was no marked increase in prevalence. Our projections indicate a substantial increase in the prevalence of diabetes to 2040. This may be partially due to the large increase in the prevalence of obesity, but also our projections for diabetes have a large uncertainty due to high item non-response in many of the surveys. In 2017, for example, every fifth case of diabetes identified by HbA_1c_ was previously not diagnosed by medical professionals.

A strength of our study is that the data used are based on population-based surveys, where samples are drawn as a random sample of the general population. In these surveys, data are collected using internationally standardised protocols and trained personnel. The response rates in these surveys are high with respect to similar surveys in other European countries [29].

Our projection method has been improved from the methods used for the estimation of previously published Finnish projections. The projection model utilises population projections and incomplete data, as well as the expected changes in influencing factors – for example the model for diabetes included obesity as a predictor. We also provided prediction intervals to demonstrate the level of uncertainty of our outcomes.

One limitation of our study was that it covered only the age group 25–64 years. Our data may also be impacted by possible selection bias by non-response, which was, however, adjusted by our multiple imputation model. Although our projection model included the population projections, their uncertainty was not available and was not included. Projections were based on previously observed developments, but we were not able to include the possible impact of public health measures or changes in society to our model.

Projections of NCD risk factors are needed to guide public health policies and programmes and to allocate health care resources for both prevention and care. They are also valuable in the communication of these topics to the public through the media. A future increase in individual risk factors may result in a substantial increase in disease incidence at the population level. For example, it has been shown in Finland during a 10-year follow-up that overweight people have a three times higher risk of diabetes than normal-weight people [30]. In further studies, risk factor projections could be translated to project disease incidence. Systematic monitoring of these risk factors among the general population through health surveys using standardised protocols is required to be able to produce reliable risk factor projections.

## Supplemental Material

sj-docx-1-sjp-10.1177_14034948221110025 – Supplemental material for Development of non-communicable disease risk factors in Finland: projections up to 2040 Click here for additional data file. Supplemental material, sj-docx-1-sjp-10.1177_14034948221110025 for Development of non-communicable disease risk factors in Finland: projections up to 2040 by Hanna Tolonen, Jaakko Reinikainen, Zhi Zhou, Tommi Härkänen, Satu Männistö, Pekka Jousilahti, Laura Paalanen, Annamari Lundqvist and Tiina Laatikainen in Scandinavian Journal of Public Health
