# Supplementary material for: Development of non-communicable disease risk factors in Finland: projections up to 2040
Source: Scand J Public Health. 2022 Jul 26;51(8):1231–8. doi: 10.1177/14034948221110025 (PMC10642220; doi:10.1177/14034948221110025)
Supplement: sj-docx-1-sjp-10.1177_14034948221110025 – Supplemental material for Development of non-communicable disease risk factors in Finland: projections up to 2040 [file sj-docx-1-sjp-10.1177_14034948221110025.docx]

**Supplementary table 1. Number (N) of respondents and participants, and item response rates (%) for used data items by sex and survey year**

|  | | **Survey year** | | | | |
| --- | --- | --- | --- | --- | --- | --- |
|  |  | **1997** | **2002** | **2007** | **2012** | **2017** |
| **Respondents (N) to the questionnaire** | **Men** | **3395** | **3247** | **2412** | **2276** | **991** |
|  | **Women** | **3763** | **3763** | **2821** | **2660** | **1079** |
| **Participants (N) to the health examination** | **Men** | **3395** | **2941** | **2242** | **2059** | **849** |
|  | **Women** | **3763** | **3490** | **2624** | **2389** | **954** |
| **Smoking question (%)** | **Men** | **99.4** | **99.8** | **99.8** | **99.8** | **95.5** |
|  | **Women** | **99.2** | **99.9** | **99.8** | **99.9** | **98.1** |
| **Sedentary lifestyle question (%)** | **Men** | **98.9** | **98.9** | **99.5** | **99.6** | **98.2** |
|  | **Women** | **98.2** | **99.2** | **99.5** | **99.4** | **98.4** |
| **Hypertension medication question (%)** | **Men** | **97.7** | **97.4** | **97.9** | **98.0** | **96.9** |
|  | **Women** | **98.4** | **98.2** | **98.1** | **98.5** | **97.6** |
| **Systolic blood pressure measurement (%)** | **Men** | **100.0** | **90.5** | **92.8** | **90.5** | **85.6** |
|  | **Women** | **99.9** | **92.7** | **92.9** | **89.7** | **88.4** |
| **Diastolic blood pressure measurement (%)** | **Men** | **100.0** | **90.5** | **92.8** | **90.5** | **85.6** |
|  | **Women** | **99.9** | **92.7** | **92.8** | **89.6** | **88.4** |
| **Cholesterol lowering medication question (%)** | **Men** | **99.5** | **99.4** | **98.7** | **99.0** | **95.8** |
|  | **Women** | **99.3** | **99.4** | **99.0** | **99.4** | **97.9** |
| **Total cholesterol measurement (%)** | **Men** | **99.6** | **90.4** | **92.5** | **90.3** | **85.5** |
|  | **Women** | **99.5** | **92.6** | **92.7** | **89.6** | **88.2** |
| **Height measurement (%)** | **Men** | **100.0** | **91.4** | **93.0** | **90.5** | **85.5** |
|  | **Women** | **99.9** | **93.0** | **93.0** | **89.8** | **88.1** |
| **Weight measurement (%)** | **Men** | **99.9** | **91.4** | **93.0** | **90.5** | **85.5** |
|  | **Women** | **99.9** | **93.0** | **93.0** | **89.8** | **88.3** |
| **Diabetes medication question (%)** | **Men** | **99.1** | **99.2** | **98.5** | **98.9** | **95.0** |
|  | **Women** | **99.0** | **99.1** | **98.9** | **99.1** | **97.7** |
| **HbA1c measurement (%)** | **Men** | **0.0** | **35.8** | **0.0** | **89.9** | **85.0** |
|  | **Women** | **0.0** | **34.4** | **0.0** | **89.0** | **88.1** |
